# Immunological changes after one year of specific immunotherapy with Pru p 3

**DOI:** 10.1186/2045-7022-5-S3-P36

**Published:** 2015-03-30

**Authors:** Francisca Gomez, Maria Jose Torres, Gador Bogas, Luisa Galindo, Tahía Fernandez, Lidia Melende, Domingo Barber, Miguel Blanca, Cristobalina Mayorga

**Affiliations:** 1 Allergy Service, Carlos Haya Hospital, Malaga, Spain; 2 Research Laboratory, Carlos Haya Hospital-FIMABIS, Malaga, Spain; 3 ALK-Abello, Madrid, Spain

## Rationale

In Southern Europe Pru p3 is the primary sensitizer of plants fruit and responsible for severe reactions. Specific immunotherapy (SIT) brings a new perspective to treat anaphylactic patients. There is a lack of knowledge regarding immunological response that includes changes in the basophil activation and antibody production during IT. We aimed to analyse early changes in the basophil response to Pru p 3 and another related allergen (Ara h 9) during the first year of sublingual immunotherapy (SLIT).

## Methods

Forty-six peach allergic patients confirmed as positive by skin prick test, ImmunoCAP IgE and/or a double blind placebo control food challenge. Basophil reactivity was determined by the basophil activation test (BAT) with Pru p 3 and Ara h 9 at three concentrations, 1, 0.1 and 0.01 µg/ml, before and after 1, 4 and 12 months of SLIT. Levels of specific IgE and IgG4 were measured at the same time intervals.

## Results

From the total number of cases 45% had anaphylaxis and 55% urticaria and/or angioedema. Sensitisation to other plants appeared in 82,6% and to pollens in 69,5%. After the first month of treatment, 28% patients showed an increase in the basophil reactivity to Pru p 3, 36% patients showed no changesin their reactivity and 36% presented a decreased reactivity to Pru p 3. Similar results were obtained for Ara h 9. We observed a decrease in IgE levels and an increase of IgG4 in the group of patients that completed a year of SIT.

## Conclusion

Preliminary results disclosed that soon after the first years of SLIT, patients showed a different behaviour in the basophil reactivity with good correlation in the response to Pru p3 and Ara h 9.After one year of SIT we observed a decrease of IgE and an increase of IgG4.

